# Tenosynovial Giant Cell Tumors of the Hand: Analysis of Risk Factors for Surgical Margin and Recurrence

**DOI:** 10.3390/medicina61060949

**Published:** 2025-05-22

**Authors:** Tolgahan Cengiz, Şafak Aydın Şimşek, Ercan Bayar, Furkan Erdoğan, Alparslan Yurtbay, Hüseyin Sina Coşkun, Ahmet Pişkin, Nevzat Dabak

**Affiliations:** 1 Clinic of Orthopedics and Traumatology, Ordu Fatsa State Hospital, Ordu 52400, Turkey; 2 Department of Orthopedics and Traumatology, Faculty of Medicine, Samsun University, Samsun 55000, Turkey; drsafakaydin@gmail.com (Ş.A.Ş.); yurtbayalparslan@gmail.com (A.Y.); 3 Clinic of Orthopedics and Traumatology, Tosya State Hospital, Kastamonu 37300, Turkey; ercannnbayarrr@gmail.com; 4 Department of Orthopedics and Traumatology, Faculty of Medicine, Ondokuz Mayis University, Samsun 55270, Turkey; erdogan27@yahoo.com (F.E.); sina.coskun@hotmail.com (H.S.C.); apiskin@omu.edu.tr (A.P.); ndabak@gmail.com (N.D.)

**Keywords:** tenosynovial giant cell tumor, hand surgery, neoplasm recurrence, soft-tissue neoplasms, risk assessment

## Abstract

*Background and Objective:* Tenosynovial giant cell tumors (TGCTs) are benign but potentially aggressive soft-tissue tumors, most commonly affecting the hand and frequently associated with local recurrence despite surgical treatment. While positive surgical margins are recognized as the strongest predictor of recurrence, the preoperative identification of factors influencing margin status remains underexplored. This study analyzed the risk factors associated with surgical margin positivity and local recurrence in patients treated for localized hand TGCTs, contributing to more accurate preoperative risk stratification. *Materials and Methods:* A retrospective analysis was conducted on 44 patients diagnosed with localized TGCTs of the hand and treated surgically at a tertiary regional hospital between 2009 and 2023. Demographic characteristics, tumor size and location, anatomical relationships (bone, joint, and neurovascular proximity), Al Qattan classification, and surgical outcomes were recorded. Binary logistic regression was used to evaluate the impact of these variables on surgical margin status and recurrence. Postoperative satisfaction was assessed using a four-choice questionnaire. *Results:* The mean patient age was 47.5 years, with 68.2% being female. The most common tumor site was the second finger (31.8%), and 20.5% of patients had positive surgical margins. Recurrence occurred in four patients (9.1%). Bone invasion, interphalangeal joint proximity, neurovascular involvement, and Al Qattan type 2 tumors were statistically significant risk factors for both surgical margin positivity and recurrence. Lesions with periosteal involvement, however, did not significantly impact recurrence risk. Among patients with positive margins, 44.4% developed recurrence. *Conclusions:* Complete surgical excision with clean margins remains the cornerstone of TGCT management. This study uniquely identifies preoperative predictors of margin positivity—key contributors to recurrence—highlighting the importance of meticulous surgical planning in high-risk cases. Close postoperative follow-up is essential, particularly for patients with positive margins, to detect and manage recurrence promptly.

## 1. Introduction

Tenosynovial giant cell tumors (TGCTs) are generally slow-growing soft-tissue tumors with a variable growth period [1]. Many names have been used to describe this type of tumor, including tenosynovial giant cell tumor, giant cell tumor of the tendon sheath, pigmented villonodular synovitis, fibrous xanthoma, benign synovioma, and sclerosing hemangioma [2]. Although the exact mechanism underlying the formation of TGCTs is still unknown, it is the most widely recognized theory explaining the pathophysiology of reactive or regenerative hyperplasia combined with an inflammatory response [3,4,5]. Recent studies have shown that this tumor exhibits chromosomal translocations involving chromosome 1p13 [6].

After ganglion cysts, TGCTs are the second most common soft-tissue tumor of the hand; 85% of cases involve the flexor tendon sheath [4]. This tumor has two histopathological subtypes: localized and diffuse. The localized type is often seen in the hand [7]. Although these tumors are more common in women, they generally affect patients between the ages of 30 and 50 [8]. Patients usually present to the clinic with a painless, slow-growing mass on the volar surface of the fingers [4,9]. The most useful diagnostic technique is magnetic resonance imaging (MRI), which is also required during the preoperative phase of preparation [10]. MRI is also helpful when using the Al Qattan classification in TGCTs. Type 1 of this classification refers to a single round or multilobed tumor, whereas type 2 refers to two or more spatially isolated tumors [4,11].

Although TGCTs are benign tumors, they have a high risk of recurrence, with a local recurrence rate varying between 7% and 44%, according to published articles [5,11,12,13]. Marginal excision with clean margins should be preferred in this tumor. Incomplete excision (surgical margin positivity) is the most definitive known risk factor for recurrence. Furthermore, cortical erosion in the surrounding bone, proximity to the thumb and other fingers’ interphalangeal joints, the existence of degenerative joint disease, Al Qattan type 2 tumors, tumors with increased mitotic activity, and the need for neurovascular dissection during excision are other known risk factors for local recurrence [5,11,12,13,14]. This study aims to determine surgical margin positivity and risk factors affecting recurrence in patients with local TGCTs of the hand and to help surgeons predict high-risk cases more accurately before surgery by revealing preoperative parameters that predict surgical margin positivity.

## 2. Materials and Methods

A retrospective study was conducted on 44 patients who were operated on with a diagnosis of TGCT localized in the distal part of the wrist joint and treated in a tertiary regional hospital between 2009 and 2023. Demographic data, preoperative radiological imaging, operation notes, and clinical examination notes of the patients were accessed from the hospital archive records. The patients’ age, gender, follow-up period, anatomical location and size of the tumor, the relationship of the tumor with the bone joint and neurovascular structures, positive surgical margins after excision, and whether there was a recurrence were recorded. MRI and operation records were considered when using the Al Qattan classification. In the positioning of the tumor, from proximal to distal, according to the broadest part of the tumor involvement, metacarpal, metacarpophalangeal joint, proximal phalanx, proximal interphalangeal joint, middle phalanx, distal interphalangeal joint, and distal phalanx were determined as anatomical points. The interphalangeal joint for the thumb was considered a separate joint. The location of the lesion relative to the volar, dorsal, and lateral aspects of the hand was also recorded. Using MRI and operation records, the relationship of the lesion with other anatomical structures was determined, and the effect of this relationship on surgical margins and recurrence was examined.

In all cases, care was taken to remove the tumor in one piece, including its capsule, during the operation. Surgical procedures were performed under 2.5× magnifying surgical loupes to enhance visualization of the tumor borders and adjacent neurovascular structures. After the operation, all patients were discharged after starting the rehabilitation process on the first postoperative day, and a clinical check-up was performed in the second week by removing the stitches. In addition, the post-operative satisfaction of these patients was tested with a 4-choice question during their follow-up.

This study has a retrospective design, which inherently carries the risk of selection and information biases. Although all patients were treated in the same center using standardized surgical protocols, some clinical data could not be fully retrieved during the retrospective review of hospital records. Specifically, preoperative radiographs were unavailable for some patients, so bone invasion was assessed solely based on MRI findings. Furthermore, due to missing documentation in patient files, the analysis did not include early postoperative complication data, such as infection and neurovascular injury.

### Statistical Analysis

Data analysis was performed using IBM SPSS V23. Data that were classified were compared using Fisher’s exact test. Binary logistic regression investigated the risk factors influencing positive surgical margins and recurrence. The analysis findings were displayed as mean ± standard deviation, median (minimum–maximum) for quantitative variables, and frequency (%) for categorical variables. A significance threshold of *p* < 0.05 was used.

## 3. Results

Of the 44 patients included in the study, 14 (31.8%) were male and 30 (68.2%) were female. The average age of the patients at the time of treatment was 47.5 ± 15.86 years (range 9 years–81 years). The average follow-up period is 69.05 ± 30.66 months (range 23 months–120 months). The lesion was most commonly located in the second finger (31.8%), and the volar face of the fingers was most commonly involved (72.7%). Anatomically, the most common location was in the proximal phalanx (25%). While the F2 zone was most frequently involved in tumors in the flexor zone, there was no dominant zone in tumors in the extensor zone. In 39 patients, the dominant hand was on the right side, 22 (50%) lesions were on the right side, and 22 (50%) were on the left. The tumors’ average size was 2.01 ± 0.84 cm (range 0.7 cm–6 cm). Descriptive statistics of the patients are shown in Table 1, and detailed anatomical locations of the lesions are shown in Table 2.

Considering the studies in the literature, the relationship between the factors associated with recurrence and the lesions was examined in detail. Accordingly, surgical margins were found to be positive in 9 (20.5%) of the 44 patients in the study. There was a periosteal relationship in 12 lesions (27.3%), and bone invasion was also detected in six patients (13.6%). Seven lesions (15.9%) were detected with close relationships with neurovascular structures. The interphalangeal joint proximity was positive in 20 patients (45.5%). Finally, according to the Al Qattan classification, the tumor was classified as type 1 in 33 patients (75%) and type 2 in 11 patients (25%) (Table 3). In our study, there were four patients (9.1%) in whom recurrence was detected. When these four patients were examined in detail, the first recurrence case was a 1.3 cm diameter tumor seen in a 35-year-old female patient located in the fifth finger volar and proximal to the distal interphalangeal joint. The patient refused reoperation and is still being followed up. The second recurrence occurred in a 58-year-old female patient, third finger lateral. It was a tumor with a diameter of 1.7 cm located in proximity to the distal interphalangeal joint. The third recurrence case was a tumor with a diameter of 2.2 cm, located in the second finger volar and in proximity to the proximal interphalangeal joint, seen in a 40-year-old female patient. The last case was a 1.5 cm diameter tumor located in the fourth finger volar and in proximity to the distal interphalangeal joint, seen in a 41-year-old female patient. These three patients were re-operated after recurrence was detected, and their follow-up continues.

**Figure 1:**
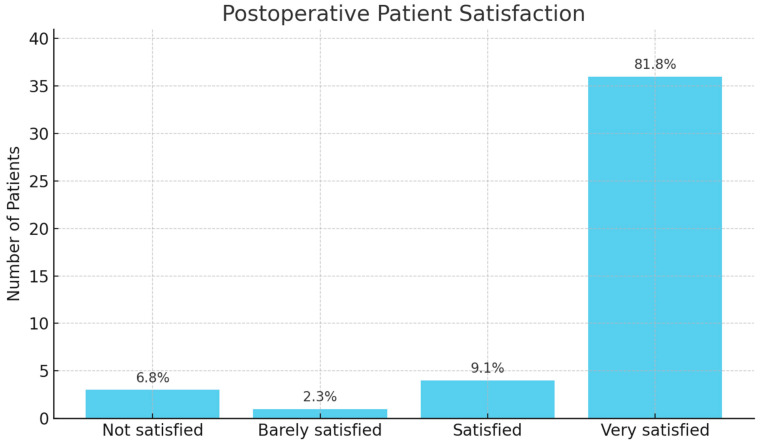
Distribution of postoperative patient satisfaction levels following surgical treatment of localized tenosynovial giant cell tumors (TGCTs) of the hand. Satisfaction was assessed using a four-choice patient-reported outcome measure during follow-up.

Risk factors affecting recurrence and surgical margin positivity were examined statistically. Accordingly, while the presence of bone invasion, proximity to the interphalangeal joint, positive surgical margins, and Al Qattan type 2 tumors were significant risk factors for recurrence, the lesion related to the periosteum did not affect recurrence (Table 4). The close relationship of the lesion with the neurovascular structures was found to be a significant parameter in both the positive surgical margin and the risk of recurrence (Table 5). Although studies frequently discuss risk factors affecting recurrence, the risk factors affecting positive surgical margins, which is the most definitive risk factor accepted for recurrence, have not been examined. In our study, the effect of risk factors on recurrence on surgical margin positivity was analyzed statistically, and periosteum-related tumors, proximity to the interphalangeal joint, presence of bone invasion, close relationship of the lesion with neurovascular structures, and tumors classified as Al Qattan type 2 were found to be significant risk factors for positive surgical margins (Table 6). Finally, the postoperative satisfaction of these patients was tested with a four-choice question during their follow-up (Figure 1). Accordingly, three patients (6.8%) answered “not satisfied”, one patient (2.3%) answered “barely satisfied”, four patients (9.1%) answered “satisfied”, and 36 patients (81.8%) answered “very satisfied”.

## 4. Discussion

TGCTs were first described as fibrous xanthoma in 1952 [4]. Many names have been used to describe this tumor, including tenosynovial giant cell tumor, giant cell tumor of the tendon sheath, pigmented villonodular synovitis, fibrous xanthoma, benign synovioma, and sclerosing hemangioma [2]. TGCTs are benign tumors and are the second most common tumor after hand ganglion cysts [15,16]. Differential diagnoses include neurofibroma, pyogenic granuloma, desmidoma, and malignant fibrous histiocytoma [4]. The diagnosis of these tumors is based mainly on a clinical examination [17]. Many previous studies have found that these hand tumors occur predominantly in women [18,19]. It is generally more common between the third and fifth decades of life [8,11]. In our study, it was more common in women than men, and the average age of the patients at the time of surgical treatment was 47.5 ± 15.86 years, which was found to be compatible with the literature. In previous studies, TGCTs were seen more frequently in the second finger of the hand but less frequently in the thumb and third finger [11,17,20]. In our study, the lesion was most commonly located on the second finger (31.8%). We also paid attention to which zone the tumor was in and which finger it was located in because we followed a postoperative rehabilitation plan according to the zone it was located in.

Although there is a large body of literature about treating TGCTs, it is still a challenging issue for surgeons. Due to the nature of the tumor, the fact that it can invade bones and joints, extend into tendon sheaths, and be surrounded by neurovascular structures are the biggest obstacles to aggressive treatment in surgery. As a result of these difficulties, recurrences can occur, and according to the literature, it is a tumor with a high risk of recurrence, with a local recurrence rate varying between 7% and 44% [5,11,12,13]. In this study, four patients (9.1%) had recurrence. Among the known risk factors for recurrence, location in the interphalangeal joint and location in the thumb are associated with recurrence [1,3,5,21]. In an exemplary study, Reilly et al. found that the risk of recurrence was increased due to TGCTs being localized in the interphalangeal joints [5]. In another study, Williams et al. found that tumors located in the thumb had a risk of recurrence [10]. In contrast to these two studies, Fotiadis et al. argued that a specific finger or phalanx was not associated with a higher risk of recurrence [3].

While investigating the risk factors that are effective in recurrence, it has been shown that tumors associated with bone, joint, and neurovascular structures relapse more frequently [1,3,5,11,21,22]. In a study conducted by Al Qattan and colleagues on this subject, it was found that intraosseous invasion increases the risk of recurrence in TGCTs [11]. In a similar study, Reilly et al. also stated that bone invasion effectively reduces recurrence [5]. Kitagawa et al., who showed that a close relationship with neurovascular structures, which is considered to be another risk factor, also increases recurrence rates, stated that the relationship with neurovascular structures is an effective risk factor because it makes excision of the tumor difficult [23]. In another study, DiGrazia and colleagues showed that the involvement of neurovascular structures was effective in recurrence [21]. Contrary studies are also available in the literature. Özben et al., contrary to these studies, argued that joint proximity and relationship with neurovascular structures do not increase the risk of recurrence [14]. Finally, some opinions accept that Al Qattan classification type 2 tumors, which are used to classify these tumors, also increase the risk of recurrence [3,11,21]. Our study evaluated the effect of these known risk factors on recurrence. Accordingly, while the bone invasion of the tumor, proximity to the interphalangeal joint, positive surgical margins, close relationship with neurovascular structures, and Al Qattan type 2 tumors are significant risk factors for recurrence, we found that the lesion’s relationship with the periosteum does not affect recurrence.

The most effective and definitive method to prevent recurrences is complete surgical excision [1,3,11,24,25]. Positive surgical margins resulting from incomplete excision are a general risk factor accepted by the authorities regarding recurrence [26]. Like many studies, our study analyzed risk factors effective in recurrence. However, unlike the literature, we wanted to investigate the risk factors that may affect surgical margin positivity and reveal which risk factors can be predicted to be positive in the presence of surgical margins during surgical planning and, therefore, which cases may be risky in terms of recurrence. We found that periosteum-related tumors, proximity to the interphalangeal joint, presence of bone invasion, close relationship of the lesion with neurovascular structures, and tumors classified as Al Qattan type 2 are significant risk factors for positive surgical margins. Our study found recurrence in four of nine cases with positive surgical margins. We did not use postoperative radiotherapy, which is recommended for these tumors in case of positive surgical margins but is not widely accepted in our cases [1,3,8,21,27]. We closely follow patients with positive surgical margins, clinically and radiologically, and recommend reoperation to patients who develop recurrence during follow-up.

In addition, the importance of technology-based approaches developed in recent years in the surgical planning of soft-tissue tumors located in anatomically limited and complex areas has been increasing. Recent studies have shown that advanced 3D planning and patient-specific guides in orthopedic oncology provide higher surgical precision in tumor excision and implant placement. Although these technologies are generally applied in the surgery of bone structures, they can also significantly contribute to the management of soft-tissue tumors. Russo et al. demonstrated the effectiveness of this approach in corrective osteotomy and prosthesis applications in proximal humerus deformities. They showed that individualized surgical strategies, precise resections, and patient-centered care can also be applied in soft-tissue surgery [28]. Such innovative planning methods have the potential to both reduce positive surgical margins and reduce recurrence rates by facilitating complete tumor removal, especially in narrow surgical areas such as the hand.

This study contributes to predicting high-risk cases in the preoperative period by comprehensively revealing the risk factors affecting surgical margin positivity for the first time in the literature, which is the most significant determinant of recurrence in tenosynovial giant cell tumors. The most important strength of our study is that all patients were evaluated and treated in the same health center with standard surgical protocols and a homogeneous treatment approach. This eliminated the variability that may arise from surgical technique differences, increased the reliability of the data obtained, and ensured that the analyzed risk factors were presented more accurately without being confused with nonsurgical variables. In addition, significant results were achieved in terms of both surgical margin positivity and recurrence thanks to the long-term follow-up of the patients. Our study has some limitations. In this study, which was designed primarily retrospectively, no information could be obtained about the functional status of the patients in the preoperative and early postoperative periods. Another limitation is that preoperative radiographs were not available for some patients. The relationship between the tumor and the bone was evaluated only with MRIs of these patients. Finally, records of complications such as infection and neurovascular injury encountered in the early postoperative period could not be accessed and were excluded from evaluation.

## 5. Conclusions

Complete excision with clean surgical margins remains the gold-standard treatment for TGCTs. This study not only confirms that positive surgical margins are the most critical predictor of recurrence but also identifies key preoperative risk factors associated with margin positivity—namely bone invasion, proximity to interphalangeal joints, close relationship with neurovascular structures, and Al Qattan type 2 classification. Recognizing these factors preoperatively allows for more informed surgical planning and risk stratification. Patients with positive surgical margins should be closely monitored clinically and radiologically, and timely reoperation should be considered in the event of recurrence.

## Figures and Tables

**Table 1:** Descriptive statistics of the patients. “Dominant side” refers to the patient’s dominant hand, and “Direction” refers to the side where the tumor is located. The term “Zone” refers to the flexor (F) or extensor (E) tendon regions of the hand.

	Frequency	%
Sex		
Male	14	31.8
Female	30	68.2
Dominant side		
Right	39	88.6
Left	5	11.4
Direction		
Right	22	50
Left	22	50
Location		
First finger	9	20.5
Second finger	14	31.8
Third finger	12	27.3
Fourth finger	5	11.4
Fifth finger	4	9.1
Zone		
E-1	1	2.3
E-2	1	2.3
E-t4	1	2.3
F-1	9	20.5
F-2	22	50
F-3	2	4.5
F-t1	3	6.8
F-t2	5	11.4
	Mean ± S. Deviation	Median (Min–Max)
Age (year)	47.5 ± 15.86	50.5 (9–81)
Follow-up (months)	69.05 ± 30.66	69 (23–120)
Tumor size (cm)	2.01 ± 0.84	1.9 (0.7–6)

**Table 2:** Descriptive statistics according to anatomical location of the tumor (dip: distal interphalangeal joint; Ip: thumb interphalangeal joint; pip: proximal interphalangeal joint; mcp: metacarpophalangeal joint).

	Anatomical Location							
	Dip	Distal Phalanx	Ip	Mcp	Metacarpal	Middle Phalanx	Pip	Proximal Phalanx
Location on finger								
Dorsal	0 (0)	0 (0)	0 (0)	0 (0)	1 (25)	1 (50)	0 (0)	0 (0)
Lateral	4 (50)	2 (66.7)	0 (0)	1 (25)	0 (0)	0 (0)	2 (20)	1 (9.1)
Volar	4 (50)	1 (33.3)	2 (100)	3 (75)	3 (75)	1 (50)	8 (80)	10 (90.9)

**Table 3:** Descriptive statistics of surgical margin positivity, recurrence, and the presence of risk factors.

	Frequency	%
Surgical margin		
Negative	35	79.5
Positive	9	20.5
Recurrence		
Positive	4	9.1
Negative	40	90.9
Periosteal relationship		
Positive	12	27.3
Negative	32	72.7
Relationship with neurovascular structures		
Positive	7	15.9
Negative	37	84.1
Bone invasion		
Positive	6	13.6
Negative	38	86.4
Proximity to interphalangeal joint		
Positive	20	45.5
Negative	24	54.5
Al Qattan classification		
Type 1	33	75
Type 2	11	25

**Table 4:** Analysis of risk factors affecting recurrence. A “Positive” value indicates the presence of the relevant feature; a “Negative” value indicates its absence. *p* < 0.05 indicates a statistically significant difference.

	Recurrence		*p* *
	Positive	Negative	
Bone invasion			
Positive	4 (66.7)	2 (33.3)	**<0.001**
Negative	0 (0)	38 (100)	
Proximity to interphalangeal joint			
Positive	4 (20)	16 (80)	**0.036**
Negative	0 (0)	24 (100)	
Surgical margin			
Negative	0 (0)	35 (100)	**0.001**
Positive	4 (44.4)	5 (55.6)	
Periosteal relationship			
Positive	3 (25)	9 (75)	0.056
Negative	1 (3.1)	31 (96.9)	
Al Qattan classification			
Type 1	1 (3)	32 (97)	**0.043**
Type 2	3 (27.3)	8 (72.7)	

* Fisher’s exact test.

**Table 5:** Effect of a close relationship with neurovascular structures on recurrence and positive surgical margins (a “positive” relationship indicates close contact with these structures during surgery).

	Relationship with Neurovascular Structures		*p* *
	Positive	Negative	
Recurrence			
Positive	4 (57.1)	0 (0)	**<0.001**
Negative	3 (42.9)	37 (100)	
Surgical margin			
Negative	0 (0)	35 (100)	**<0.001**
Positive	7 (77.8)	2 (22.2)	

* Fisher’s exact test.

**Table 6:** Analysis of risk factors affecting positive surgical margins. A “Positive” value indicates the presence of the relevant feature; a “Negative” value indicates its absence. *p* < 0.05 indicates a statistically significant difference.

	Surgical Margin		
	Negative	Positive	*p* *
Al Qattan classification			
Type 1	29 (87.9)	4 (12.1)	**0.026**
Type 2	6 (54.5)	5 (45.5)	
Relationship with neurovascular structures			
Positive	0 (0)	7 (100)	**<0.001**
Negative	35 (94.6)	2 (5.4)	
Periosteal relationship			
Positive	6 (50)	6 (50)	**0.007**
Negative	29 (90.6)	3 (9.4)	
Proximity to interphalangeal joint			
Positive	13 (65)	7 (35)	**0.042**
Negative	22 (91.7)	2 (8.3)	
Bone invasion			
Positive	2 (33.3)	4 (66.7)	**0.009**
Negative	33 (86.8)	5 (13.2)	

* Fisher’s exact test.

## Data Availability

All data can be transmitted to the journal if requested.
